# Leptospirosis in Germany, 1962–2003

**DOI:** 10.3201/eid1107.041172

**Published:** 2005-07

**Authors:** Andreas Jansen, Irene Schöneberg, Christina Frank, Katharina Alpers, Thomas Schneider, Klaus Stark

**Affiliations:** * Robert Koch Institute, Berlin, Germany;; † Charité, Berlin, Germany

**Keywords:** leptospirosis, Leptospira interrogans, vector-borne diseases, epidemiology, developed countries, Germany

## Abstract

Epidemiologic trends of human leptospirosis in Germany were investigated by analyzing national surveillance data from 1962 to 2003 and by conducting a questionnaire-based survey from 1997 to 2000. After a steady decrease of leptospirosis incidence from 1962 to 1997, surveillance data indicate an increase in disease incidence to 0.06 per 100,000 (1998–2003). Of 102 laboratory-confirmed cases in humans from 1997 to 2000, 30% were related to occupational exposures. Recreational exposures were reported in 30% (including traveling abroad in 16%), whereas residential exposure accounted for 37% of the cases. Direct contact with animals, mostly rats and dogs, was observed in 31% of the cases. We conclude that recent changes in transmission patterns of leptospirosis, partially caused by an expanding rat population and the resurgence of canine leptospirosis, may facilitate the spread of the disease in temperate countries like Germany. Preventive measures should be adapted to the changing epidemiology of leptospirosis.

Leptospirosis is a zoonotic disease of global importance, caused by spirochetes of the genus *Leptospira*. Based on antigenic relatedness, human pathogenic *Leptospira interrogans* strains have been differentiated into >200 serovars, which are classified into 24 serogroups (1). A variety of wild and domestic animals form the natural reservoir for pathogenic leptospires. Some serovars are associated with specific hosts, like *L. interrogans* serovar Icterohaemorrhagiae, which is primarily harbored by rats. Transmission to humans results from exposure to the urine of infected animals either by direct contact or through contaminated soil or water. Leptospirosis has recently been classified as a reemerging infectious disease, particularly in tropical and subtropical regions (2,3). Though the infection is considered rare in developed countries, low but persisting rates of autochthonous illness and death exist. Due to its nonspecific clinical features, a lack of awareness among physicians, and difficulties in isolating the organism and serologic testing, the incidence of leptospirosis is likely underestimated (4). Recent seroepidemiologic studies suggest that the incidence of leptospirosis in urban centers of some industrialized countries is remarkably high, with seroprevalence of leptospiral antibodies reaching levels ≥30% in selected urban populations (5–9). In Germany, the documented history of leptospirosis began in 1886 when Adolf Weil first described the severe form of the disease (10). Large agriculture-associated epidemics with several thousands of infected persons occurred from the 1920s to the 1960s (11). Although human leptospirosis is endemic in most industrialized countries worldwide (12), few systematic large-scale reports provide detailed information on the epidemiologic characteristics of the disease in temperate regions, including data on the relationship between humans and potential reservoir hosts and urban versus rural settings. However, this information is essential to implement appropriate control measures. The objective of this study is to describe current trends of laboratory-confirmed human leptospirosis in Germany, with special focus on modes of transmission, implicated reservoir hosts, prevalent serovars, and regional distribution of the infection.

## Methods

Leptospirosis was a reportable disease in the former German Democratic Republic after 1958. In the former Federal Republic of Germany, cases of leptospirosis were reportable under the Federal Communicable Disease Act since 1962. After the reunification in 1990, the 2 reporting systems were combined. Hospitals and outpatient facilities reported cases of leptospirosis to the local health departments and through state health authorities to the Robert Koch Institute. In 2001, an improved surveillance system for mandatory case reporting of infectious diseases was implemented in Germany. Under the Infectious Disease Control Act, German laboratories notify local public health authorities of positive test results for leptospirosis. Local authorities then obtain additional information on the patient and electronically transfer the data through state authorities to the central database at the Robert Koch Institute. Additionally, official statistical data on deaths due to leptospirosis that were documented on death certificates (1962–2002) were obtained from the Federal Office of Statistics (http://www.gbe-bund.de).

From 1997 to 2000, data on demographics (age, sex, and residence), onset of symptoms, country in which infection was contracted, possible exposure risks, infecting serovars, mortality, and causes of death were evaluated by standardized questionnaires sent to local health departments for every reported case of leptospirosis. From 2001 to 2003, data on demographics, onset of symptoms, and country in which the infection was contracted were transmitted to the Robert Koch Institute as required by the newly implemented Infectious Disease Control Act. A case definition, demanding both clinical signs of leptospirosis (at a minimum, acute febrile illness) and laboratory confirmation (positive culture, polymerase chain reaction, seroconversion, or a single significant antibody titer as demonstrated by enzyme-linked immunosorbent assay, complement fixation testing, or microscopic agglutination testing), was applied to all reported infections from 1997 to 2003 (13). Identification of the presumptive infecting leptospiral serovar was performed either by culture (7 cases) or microscopic agglutination testing (38 cases) in laboratories with personnel who were experienced in these types of procedures. In 7 cases, a serovar-specific complement fixation test was conducted. All cases with documented cross-reactivity between different leptospiral serovars were excluded from the analysis.

To assess temporal trends, mean annual incidences were calculated for 6-year intervals from 1962 to 2003, referenced to the population of year 4 of each interval. Incidences for each of the 16 German states from 1997 to 2003 were calculated similarly. Statistical tests for trend were performed with the extension of the Mantel-Haenszel chi-square test. Data were analyzed with EpiInfo, version 6.04d (Centers for Disease Control and Prevention, Atlanta, GA, USA). The Mann-Whitney test was used to compare quantitative variables. A p value of <0.05 was considered to be significant.

## Results

**Figure 1:**
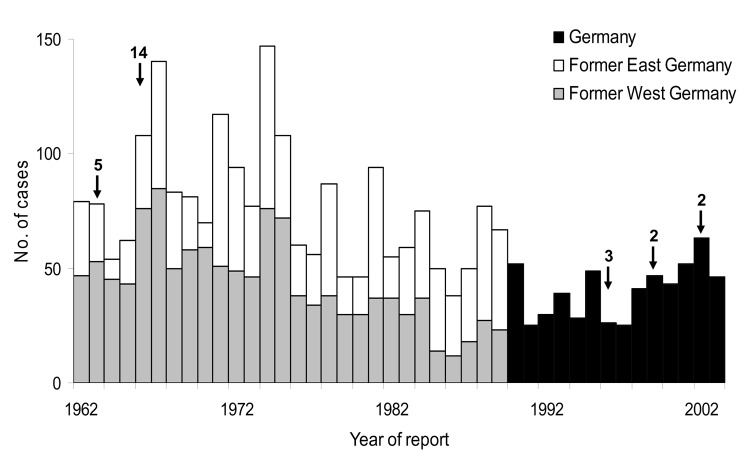
Number of reported leptospirosis cases in Germany, 1962–2003. Arrows indicate outbreaks and number of cases affected.

From 1962 to 2003, a total of 2,694 cases of leptospirosis were reported. During this 41-year period, the number of human leptospirosis cases generally declined, with a maximum of 147 cases in 1974 and a minimum of 25 cases in both 1991 and 1997 (median 59 cases ) (Figure 1). The mean annual incidence decreased from 0.11 per 100,000 population from 1962 to1967 to the lowest observed incidence of 0.04 per 100,000 population (p<0.001) from 1992 to 1997. From the period 1992–1997 to the period 1998–2003, incidence increased to 0.06 per 100,000 population (p = 0.001). There were 234 deaths caused by leptospirosis from 1962 to 2002 (overall case-fatality ratio 9%). The highest fatality ratio was 12% in the period 1968–1973, while the lowest fatality ratio was 5% in the period 1998–2002. Five minor outbreaks of leptospirosis were reported: 5 cases in 1963, 14 cases in 1966, 3 cases in 1996, 2 cases in 1999, and 2 cases in 2002.

**Figure 2:**
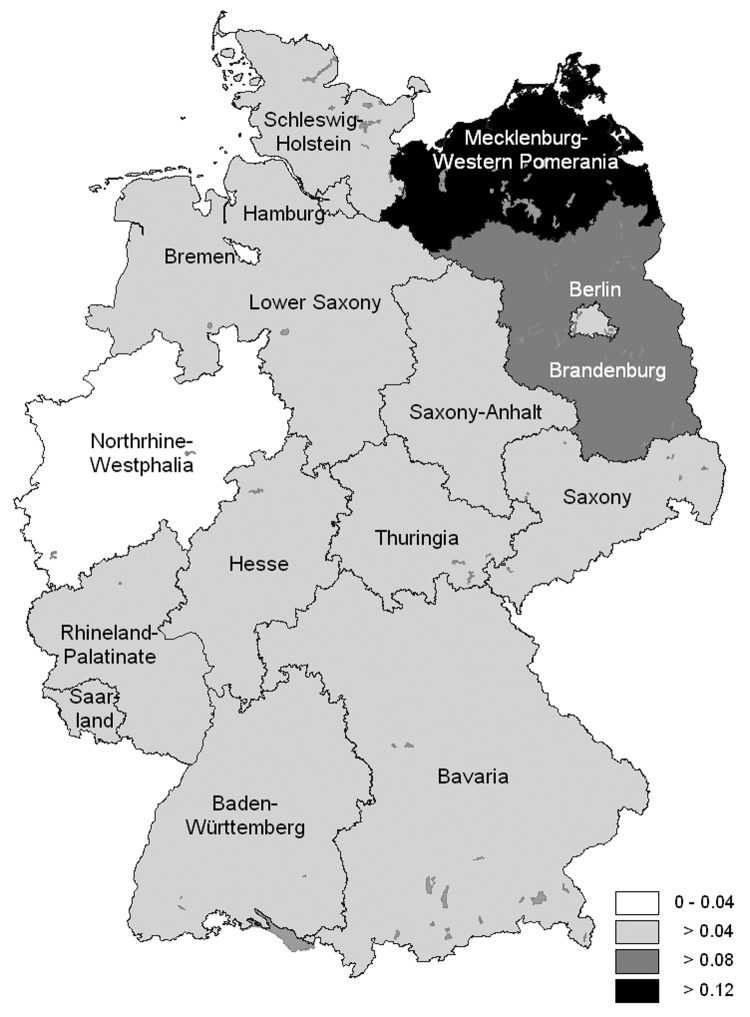
Leptospirosis cases 1997–2003, distribution by age and sex (N = 269).

**Figure 3:**
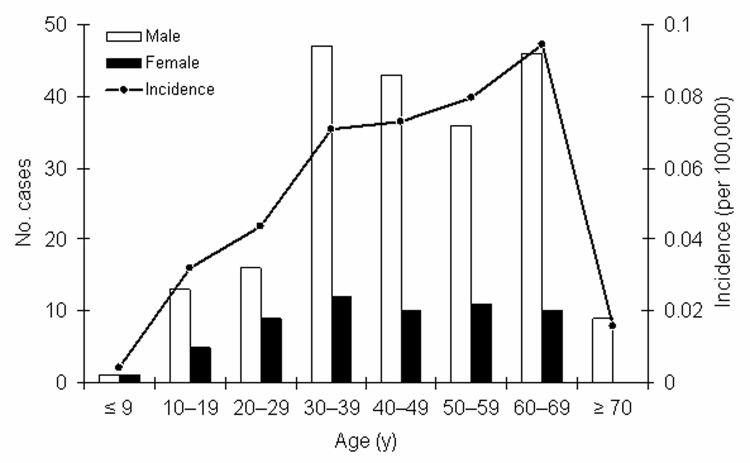
Regional distribution of leptospirosis in Germany, 1997–2003. Incidence per 100,000 population.

From 1997 to 2003, a total of 317 cases of leptospirosis were reported. Of these, 269 (85%) fulfilled the case definition and were included in the analysis. Information on age, sex, and area of residence was obtained for all 269 case-patients. Seventy-eight percent were men; 80% were 30–69 years of age (median age 45 years, range 1 month to 80 years) (Figure 2). Age-specific incidence was highest for persons 60–69 years of age, with a mean annual incidence of 0.1 per 100,000, and lowest incidence for children <10 years of age with 0.004 per 100,000 (Figure 2). Incidence was highest in the northeastern states of Germany (Mecklenburg-Western Pomerania with 0.12 per 100,000 population, followed by Brandenburg with 0.09 per 100,000 population) (Figure 3).

**Figure 4:**
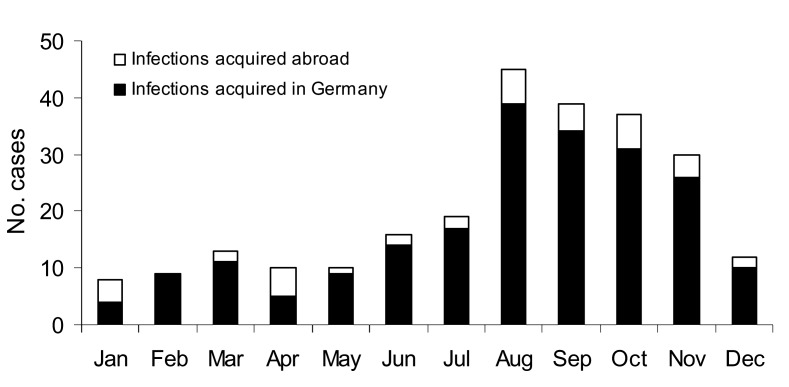
Seasonal trends of leptospirosis, 1997–2003 (N = 248).

Presumed date and country in which the infection was contracted were reported for 248 cases (92%). In 39 cases (16%), the infection was likely contracted outside Germany. Of these, 13 patients named destinations inside Europe (4 cases from France, 2 cases each from Greece, Poland, and Hungary, and 1 case each from Norway, Croatia, and Bulgaria). Non-European countries were named in 26 cases, including 3 cases each from the Dominican Republic, Mexico, and Thailand, 2 cases each from Cuba and Argentina, and 1 case each from Vietnam, Jamaica, Australia, Argentina, Bahamas, Costa Rica, India, Indonesia, Japan, Venezuela, Kenya, Turkey, and the Russian Federation. In most cases, onset of disease was August–November (61% in total, 62% of autochthonous cases, and 54% of imported cases) (Figure 4).

From 1997 to 2000, local health authorities returned 140 completed questionnaires for 156 reported case-patients (response rate of 90%). Of these, 14 (10%) did not fulfill the case definition and were excluded from analysis. Information on type of exposure was available for 102 (81%) of the remaining 126 cases. Occupational and recreational exposure risks were both present in 30% of the patients. Accidental exposures (falls into water, animal bites) were documented in 3%, while 37% of the exposures occurred through residential activities (Table). Traveling abroad was the single most frequent documented exposure risk (16%). Among patients with a known travel history, exposure to fresh water during travel was reported in 6 (38%) of 16 cases.

**Table:** Type of exposure for 102 confirmed cases of leptospirosis in Germany, 1997–2000

Type of exposure	No. cases (%)
Occupational	31 (30)
Park ranger	2
Sewer worker	6
Veterinarian	1
Zoo director	1
Abattoir worker	3
Miner	1
Military	1
Waste management	1
Construction work	3
Farmer with livestock	5
Farmer without livestock	7
Recreational	31 (30)
Fishing	5
Swimming	6
Camping	1
Traveling	16
Canoeing	3
Accidental	3 (3)
Fall in water	2
Bite	1
Residential	37 (37)
Keeping animals as pets	11
Rats/mice around home	4
Mud/water around home	5
Working on private canal, ditch, pond	9
Living on farm	2
Gardening or yard work	6
Total	102 (100)

A similar distribution of exposure types was found in all age groups, except in persons ≥60 years of age, where residential exposure risks (60%) clearly dominated over recreational (16%) and occupational exposures (24%). Contact with domestic animals was reported in 12 (12%) of 102 cases, including dogs (7 cases), rabbits (2 cases), a mouse, a pet rat, and a horse (1 case each). Other animal exposures were reported in 20 cases (20%), including the following animals: rats (13 cases), cattle (3 cases), mice (2 cases), a pig, and a sheep (1 case).

Of the 102 patients with reported exposure risks, 24 (24%) lived in urban areas with 12 of those likely being infected in the cities, and 12 likely being infected during excursions or holidays outside the cities. Genuine urban cases were mainly related to residential exposures (gardening and associated activities in 6 cases) and owning dogs (4 cases). Accidental falls into urban waterways were reported in 2 cases.

A definite serovar identification was recorded in 52 (41%) of the 126 cases, while cross-reactivity between several serovars was observed in 22 cases (18%). In 52 cases (41%), no serovar differentiation was available. Serovars in the Icterohaemorrhagiae serogroup were the most common, accounting for 32 (62%) of the 52 identified isolates, followed by *L. interrogans* serovars Canicola and Grippotyphosa, each accounting for 5 (9%) of the identified isolates. Two cases (4%) with *L. interrogans* serovar Bataviae and 3 cases (6%) with serovar Pomona were identified, while serovars Sejroe, Hardjo, Hebdomadis, Bratislava, and Australis were found in single cases.

Of the 126 reported patients from 1997 to 2000, 10 patients died (90% men, case fatality 8%). The median age was 44 years of age for survivors and 60 years of age for nonsurvivors (p<0.03). Causes of death were multiple organ failure (including the triad of fever, renal failure, and jaundice) in 6 cases and massive pulmonary hemorrhage, intracerebral hemorrhage, cardiopulmonary failure, and acute respiratory distress syndrome in single cases. One of the nonsurvivors lived in an urban area and contracted the disease from an accidental fall into a waterway in Hamburg. Further exposure risks in the fatal cases included traveling abroad (3 patients), farming (2 patients), contact to a dog (1 patient), working in an abattoir (1 patient), mud or water around home (1 patient), and sewage work (1 patient). Serovars were identified in 3 of these cases (Bataviae, Icterohaemorrhagiae, and Canicola).

## Discussion

After a steady decrease in the incidence of leptospirosis in Germany from 1962 to 1997, national surveillance data suggest an increased frequency of the disease in recent years. Though part of the increase in case reports since 2001 may reflect the acceptance of the improved surveillance system in Germany among laboratories that identify leptospirosis, the increase was already observable in the preceding surveillance system and likely reflects a true rise in cases. Presumably, the number of reported cases from Germany represents the tip of the iceberg, since less severe and nonspecific clinical manifestations of leptospirosis frequently go unrecognized, and several studies indicate that subclinical infection is common worldwide (14,15). As to the documented history of leptospirosis in Germany, this possible reemergence appears to be associated with distinct changes in the epidemiologic characteristics of the disease that affect routes of transmission, host animals, prevalent serovars, population at risk, and regional distribution.

In Germany, leptospirosis historically has been associated with agricultural exposure risks and mainly restricted to rural environments (11). *L. interrogans* serovar Grippotyphosa represented the most common infecting serovar, usually transmitted by field voles (*Microtus apodemus*) and European hamsters (*Cricetus cricetus*) as reservoir animals (16). Along with ecologic and structural changes, including mechanization of agriculture and improvements in sanitation, the relevance of leptospirosis in the rural economy diminished in the 1960s (17). A similar reduction in disease activity was reported from other industrialized countries, where leptospirosis has traditionally been a common occupational disease among agricultural workers (18,19). At present, some of the traditional occupations described as risks for leptospirosis (e.g., livestock farmers and sewage and abattoir workers) are still found in Germany. However, traveling abroad is the single most important exposure risk identified in the recent study. This finding is consistent with a general trend observed in several other countries, especially among the growing numbers of participants in adventure travel and water sports in exotic locations (20,21). Along with expanding long distance travel, the increase of leptospirosis as an emerging disease in tropical areas will necessarily be reflected in travelers returning from these regions.

However, the risk for exposure due to traveling is not restricted to the tropics, as 33% of the patients with a travel history contracted the infection while visiting European countries. France was unexpectedly the most common reported travel destination in this study. With an incidence of 0.44 per 100,000 population in 2000 (22), France has one of the highest reported incidences of leptospirosis in Western Europe. Leisure activities associated with exposure to fresh water (in particular, canoeing) were identified as a risk factor for the disease, and travelers to France with an interest in water sports should be aware of this association (23).

In at least 12% of the reported cases, leptospirosis was contracted in urban areas and was primarily related to residential activities, like gardening and working on private ponds. Though urban leptospirosis has been established as a threat for certain occupational groups in Germany (17), residential exposures represent a new category of urban risk factors. The emergence of sporadic urban leptospirosis has recently been reported from several other industrialized countries, including the United States and Israel (9,18,24), and our data support the view of leptospirosis as an underrecognized urban health problem in industrialized countries (9). Since leptospirosis is still perceived as a zoonotic disease mainly restricted to occupational exposures and rural areas, clinicians may fail to consider the disease in urban settings (9).

In both urban and rural environments, we found domestic animals in general, and dogs in particular, implicated as putative reservoir hosts. The growing importance of canine leptospirosis in Germany is underlined by a seroepidemiologic study conducted from 1999 to 2002. Of 3,671 canine serum samples, 29.8% showed high antibody titers against several leptospiral serovars not covered by customary vaccines, including *L. interrogans* serovars Bratislava in 4.8% and Saxkoebing in 3.2% (25). In the United States and Canada, a rising prevalence of the disease in dogs has been observed since the early 1990s (26,27), and canine leptospirosis has recently been recognized as a reemerging zoonosis (28). Aside from the possible transmission of leptospirosis through direct contact with urine of infected pets, dogs and other domestic animals kept in gardens may also increase the likelihood of concomitant rodent infestation, as rodents are attracted by food and shelter because of the presence of domestic animals (29). The chance of rat infestations in such habitats is likely to rise, corresponding to a general increase of rat populations in both urban and rural environments noted in Germany (German Federal Environmental Agency, unpub. data).

In addition to dogs and rodents, a variety of domestic and wild animals were identified as potential reservoirs for leptospires in Germany. A nationwide seroepidemiologic study of almost 31,000 different animals showed the prevalence for *Leptospira* species was 14.4% in sheep and 4.5% in horses (30). In cattle, seroprevalence for *L. interrogans* serovar Hardjo was 10.3% and for *L. interrogans* serovar Saxkoebing was 11.3% (31). Leptospiral antibodies were also detected in foxes (2% of 1,253 animals) and wild boars (24% of 245 animals) (32,33), thus demonstrating the extensive distribution of *Leptospira* species in the German fauna and, consequently, the necessity for epidemiologic studies in defined occupational groups with close contact to these animals.

As to the distribution of infecting serovars, we found that *L. interrogans* serovar Icterohaemorrhagiae had replaced *L. interrogans* serovar Grippotyphosa as the most prevalent serovar in Germany. Although serologic identification of specific serovars by using microscopic agglutination testing can only give a broad idea of the common serovars in a certain population and has to be interpreted cautiously (34), our data indicate a principal shift from agriculture-related serovars to those primarily related to nonoccupational modes of transmission, with rats as their principal vector. This change has also been observed in other European countries (19,35). Underlying changes in transmission modes may also explain the shift to higher ages of infection that we observed when compared to ages noted in other studies. In several countries with predominant recreational or occupational modes of transmission, leptospirosis mainly affects persons 20–50 years of age (35–37). Although many patients in Germany are in this age group, the highest age-specific incidence was found in persons 60–69 years of age. This shift may be explained by the observed predominance of residential exposure risks, which are largely independent of physical fitness or ability to work. The seasonal distribution of leptospirosis in Germany was found to be similar when compared to the past and to recent studies from other temperate countries (19,35), which likely reflects the sensitivity of the bacteria to climatic variation and the seasonality of transmission patterns, irrespective of the predominant serovars.

Leptospirosis was traditionally most common in German states with a pronounced rural economy, including Bavaria, Schleswig-Holstein, Lower Saxony, and Saxony (11). From 1997 to 2003, however, the highest incidence of leptospirosis was found in the eastern states of Germany, particularly in Mecklenburg-Western Pomerania. The corresponding region of the former German Democratic Republic showed a high number of human leptospirosis caused by *L. interrogans* serovar Icterohaemorrhagiae already occurring in the 1950s. It has been suggested that this high incidence was correlated with large populations of Norway rats, which were attracted by the fishing industries (38). In addition, Mecklenburg-Western Pomerania has the highest proportion of surface fresh water among the German states (excluding the city states) and is particularly rich in lakes, canals, and branched rivers, which are frequently used for recreational activities by local persons and persons from nearby cities on weekend trips. Although this region was barely affected by agriculture-related leptospirosis in the past, it apparently provides favorable conditions for rat infestations and consequent transmission of *L. interrogans* serovar Icterohaemorrhagiae to humans by means of freshwater exposure.

Consistent with the literature, we found that nonsurvivors were significantly older than survivors (39). With respect to the increased proportion of elderly persons affected by the disease in Germany, this finding is of major importance, since it suggests a possible increase in fatal courses of leptospirosis in the future. Finally, we documented the first fatal case of leptospirosis due to massive pulmonary hemorrhage observed in Germany. Pulmonary hemorrhage has increasingly been recognized as a grave manifestation of leptospirosis in several regions of Asia and South and Central America, but it has rarely been observed in central Europe (1). Since severe pulmonary involvement with leptospirosis is actually underrecognized in tropical regions of high endemicity (40), clinicians in industrialized countries are even more likely not to consider or recognize pulmonary complications in leptospirosis. Although the high case-fatality ratio demonstrated in our study may be biased toward including only the most severely ill or hospitalized patients, insufficient knowledge of the protean clinical manifestations of leptospirosis among clinicians clearly facilitates misdiagnosis and leads to delays in antimicrobial drug therapy of this potentially treatable infection.

## Conclusion

In conclusion, this study suggests that the conditions for the transmission of leptospirosis to humans have become more favorable in recent years. Increasing international travel as well as the rising popularity of recreational and residential activities linked to freshwater exposures may explain some of the observed increase. In addition, the expansion of rat populations and the resurgence of leptospirosis in dogs potentially promote establishing an endemic substrate for the spread of the disease, especially in urban regions. Since these changes in transmission biology appear not to be restricted to Germany, leptospirosis should at least be an issue of increased concern in other industrialized countries. Reliable baseline information as provided by continuing surveillance and case reporting are indispensable for implementing appropriate preventive measures. In Germany, efforts should focus on rodent control and region-specific travel advice. In addition, developing and using canine vaccines that cover the prevalent serovars should be encouraged.
